# Draft Genome Sequence of *Staphylococcus fleurettii* Strain MBTS-1 Isolated from Cucumber

**DOI:** 10.1128/genomeA.00335-17

**Published:** 2017-05-11

**Authors:** Bo Li, Erik Brinks, Charles M. A. P. Franz, Gyu-Sung Cho

**Affiliations:** Department of Microbiology and Biotechnology of the Max Rubner-Institut, Federal Research Institute of Nutrition and Food, Kiel, Germany

## Abstract

The draft genome of *Staphylococcus fleurettii* MBTS-1, isolated from cucumber in northern Germany, was sequenced. Analysis showed that the assembled genome had a size of 2,582,128 bp with a predicted total of 2,491 protein-encoding genes, 9 rRNAs, 5 ncRNAs, and 44 tRNAs. This strain did not contain plasmid DNA.

## GENOME ANNOUNCEMENT

Coagulase-negative staphylococci (CONS) are commonly isolated from food, the environment, the mucous membranes of animals, as well as from the skin of humans and animals. *Staphylococcus fleurettii* is a member of the CONS and is an oxidase-positive and generally novobiocin-resistant species (1). *S. fleurettii* naturally contains the chromosomally located *mecA* methicillin-resistance gene, which encodes penicillin-binding protein (PBP) 2a that has a low affinity for beta-lactam antibiotics (2). In methicillin-resistant *S. aureus* strains, the methicillin-resistance genes are located on a mobile staphylococcal cassette chromosome (SCC) (3). As in *S. fleurettii*, the *mec*A gene is intrinsic and is located on the chromosome; it is suspected that *S. fleurettii* is the genetic origin of *mecA* genes in other staphylococci.

The *S. fleurettii* MBTS-1 strain was isolated from a cucumber in Germany. The total genomic DNA was isolated using the peqGOLD Bacterial DNA isolation kit (Peqlab, Erlangen, Germany). The sequencing library was prepared with an Illumina Nextera XT library prep kit (Illumina, USA) and was run on an Illumina MiSeq sequencer with paired-end reads of 250 bp. In total 1,289,304 paired-end sequence reads with lengths of 250-bp were obtained with an average 127-fold coverage. The reads were *de novo* assembled using SPAdes version 3.10 (4), and the *N*_50_ was 168,305 bp. The draft genome size of *S. fleurettii* MBTS-1 was 2,582,128 bp with a mol% G+C content of 31.7. The genome sequence was annotated using the NCBI Prokaryote Genome Annotation Pipeline and the RAST genome annotation server (5, 6). The genome contained 2,491 protein-encoding genes, 56 pseudogenes, 9 rRNAs, and 44 tRNAs. This is the first draft genome sequence available for *S. fleurettii*, and the genome information for this species will be useful for evolutionary genomic analyses of SCCmec elements of methicillin-resistant staphylococci strains.

### Accession number(s).

The whole-genome shotgun project of *S. fleurettii* MBTS-1 has been deposited at DDBJ/ENA/GenBank under the accession number MWJM00000000.
